# Glyceraldehyde 3-phosphate dehydrogenase negatively regulates human immunodeficiency virus type 1 infection

**DOI:** 10.1186/1742-4690-9-107

**Published:** 2012-12-13

**Authors:** Naoki Kishimoto, Ayano Onitsuka, Keishi Kido, Nobutoki Takamune, Shozo Shoji, Shogo Misumi

**Affiliations:** 1 Department of Pharmaceutical Biochemistry, Faculty of Medical and Pharmaceutical Sciences, Kumamoto University, Kumamoto, 862-0973, Japan; 2 Kumamoto Health Science University, Kumamoto, 861-5598, Japan

**Keywords:** Glyceraldehyde 3-phosphate dehydrogenase, Lysyl-tRNA synthetase, tRNA^Lys3^, Human immunodeficiency virus type 1

## Abstract

**Background:**

Host proteins are incorporated inside human immunodeficiency virus type 1 (HIV-1) virions during assembly and can either positively or negatively regulate HIV-1 infection. Although the identification efficiency of host proteins is improved by mass spectrometry, how those host proteins affect HIV-1 replication has not yet been fully clarified.

**Results:**

In this study, we show that virion-associated glyceraldehyde 3-phosphate dehydrogenase (GAPDH) does not allosterically inactivate HIV-1 reverse transcriptase (RT) but decreases the efficiency of reverse transcription reactions by decreasing the packaging efficiency of lysyl-tRNA synthetase (LysRS) and tRNA^Lys3^ into HIV-1 virions. Two-dimensional (2D) gel electrophoresis demonstrated that some isozymes of GAPDH with different isoelectric points were expressed in HIV-1-producing CEM/LAV-1 cells, and a proportion of GAPDH was selectively incorporated into the virions. Suppression of GAPDH expression by RNA interference in CEM/LAV-1 cells resulted in decreased GAPDH packaging inside the virions, and the GAPDH-packaging-defective virus maintained at least control levels of viral production but increased the infectivity. Quantitative analysis of reverse transcription products indicated that the levels of early cDNA products of the GAPDH-packaging-defective virus were higher than those of the control virus owing to the higher packaging efficiencies of LysRS and tRNA^Lys3^ into the virions rather than the GAPDH-dependent negative allosteric modulation for RT. Furthermore, immunoprecipitation assay using an anti-GAPDH antibody showed that GAPDH directly interacted with Pr55^*gag*^ and p160^*gag*-*pol*^ and the overexpression of LysRS in HIV-1-producing cells resulted in a decrease in the efficiency of GAPDH packaging in HIV particles. In contrast, the viruses produced from cells expressing a high level of GAPDH showed decreased infectivity in TZM-bl cells and reverse transcription efficiency in TZM-bl cells and peripheral blood mononuclear cells (PBMCs).

**Conclusions:**

These findings indicate that GAPDH negatively regulates HIV-1 infection and provide insights into a novel function of GAPDH in the HIV-1 life cycle and a new host defense mechanism against HIV-1 infection.

## Background

Because the HIV-1 genome only encodes a limited number of viral proteins, HIV-1 must take advantage of multiple functions of host proteins in order to successfully replicate. Several studies of purified HIV-1 virions have shown that in addition to proteins encoded by the virus, host proteins are found in the virions [1]. Some of these proteins may be taken into the virions simply because of their proximity to the viral assembly and budding sites, while other host proteins, such as cyclophilin A and LysRS, are included in HIV-1 particles as a result of their interaction with *gag* or *gagpol* proteins during assembly [2-7]. These host proteins play an important role in facilitating the process of *gag* protein folding and tRNA^Lys3^ packaging. Therefore, one way to elucidate the viral replication capacity gained by the packaging of host proteins is to directly analyze the host proteins inside the virions. A purified HIV-1_LAV-1_ preparation was analyzed by 2D gel electrophoresis and matrix-assisted laser desorption/ionization-time-of-flight mass spectrometry (MALDI-TOF MS). Proteome analysis demonstrates that GAPDH is inside the virions.

GAPDH is a prototype “moonlighting” protein that is involved in glycolysis, the carbon reduction cycle, the exportation of nuclear RNA, DNA repair, the bundling of microtubules, and apoptosis [8-14]. Furthermore, GAPDH also regulates viral replication. Interestingly, GAPDH phosphorylates the hepatitis B virus core protein [15] and binds with the *cis*-acting RNAs of several viruses, such as the 5′ UTR of the hepatitis A virus [16], the 3′ UTR sequences of the human parainfluenza virus [17], and the hepatitis C virus [18]. These findings indicated that GAPDH might also play a role in regulating HIV-1 replication.

In this study, we show that GAPDH is incorporated into the virions and the suppression of GAPDH packaging inside the virions enhances viral infection owing to the high reverse transcription efficiency. These results elucidate the viral replication capacity gained by GAPDH packaging and reveal a novel regulation step of HIV-1 infection.

## Results

### Multiple GAPDH isozymes were incorporated into virions

**Figure 1:**
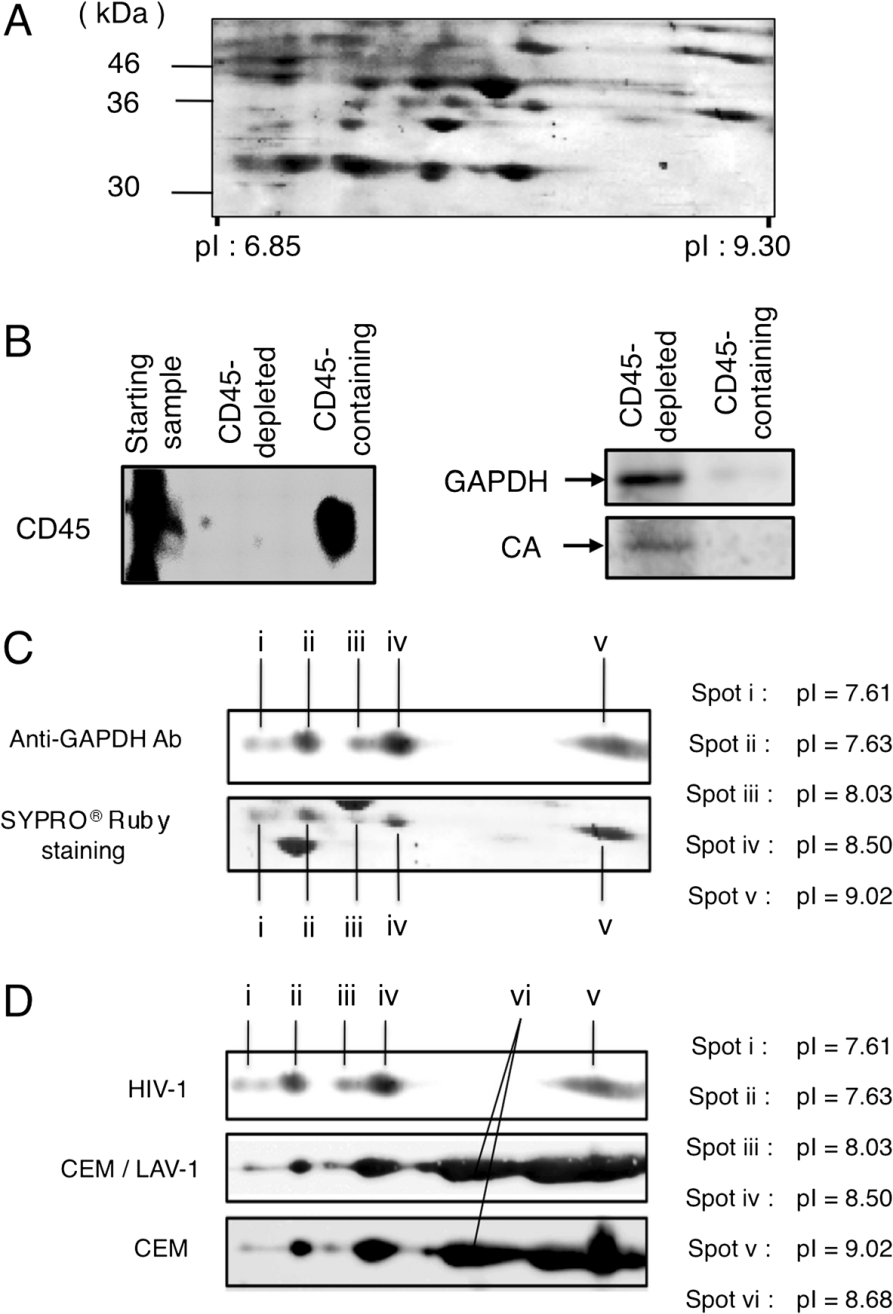
**Confirmation of GAPDH incorporation into HIV**-**1 virions.** (**A**) SYPRO® Ruby-stained 2D gel image of proteins from highly purified HIV-1_LAV-1_ virions within the pI range of 6.85-9.30 and the molecular weight range of 30–46 kDa. (**B**) Western immunoblotting of GAPDH in CD45-depleted fraction and CD45-containing fraction. CD45 and CA levels were used as the markers of microvesicles and viral particles, respectively. Samples from the CD45-immunoaffinity-depleted fraction (CD45-depleted fraction) and anti-CD45 beads fraction (CD45-containing fraction) are identified above their respective lanes. The antibodies used are indicated on the left side of each blot. (**C**) Detection of virion-associated GAPDH isozymes. The upper panel shows the results of western immunoblotting using an anti-GAPDH antibody and the lower panel indicates the corresponding SYPRO® Ruby-stained gel. (**D**) Comparison of pI values of GAPDH isozymes among HIV-1_LAV-1_ (upper panel), CEM/LAV-1 (middle panel), and CEM cells (lower panel). GAPDH isozymes were detected by western immunoblotting using the anti-GAPDH antibody. Spot pI values are shown in the figure.

To examine the host proteins found in virions produced by an HIV-infected cell line, CEM/LAV-1 cells, a highly purified HIV-1_LAV-1_ preparation was analyzed by 2D gel electrophoresis, followed by MALDI-TOF MS/MS (Figure 1A). The MS/MS ion search predicted the identity of the 36-kDa proteins to be GAPDH. To confirm whether GAPDH is incorporated into virions, contaminating particles such as microvesicles and exosomes were removed according to previously described methods [19]. Western immunoblotting for CD45, GAPDH, and the capsid (CA) protein was carried out. In the starting sample, the CD45 signal was detected (Figure 1B left panel). On the other hand, no CD45 signal was detected in the lane of the CD45-depleted fraction (flow-through fraction), indicating that most of the CD45-positive contaminating particles such as microvesicles and exosomes in the starting material were removed (Figure 1B left panel). Therefore, as shown in Figure 1B right panel, western immunoblotting for GAPDH, and the CA protein was carried out. The data showed that there were evident GAPDH and CA signals in the CD45-depleted fraction, while there is hardly any GAPDH signal in the CD45-containing fraction, indicating that GAPDH from CEM/LAV-1 cells may exclusively be incorporated into the virions (Figure 1B right panel). Therefore, unless otherwise noted, HIV-1 preparations were systematically prepared without the CD45 depletion procedure in the following study. To gain further insight into the incorporation of GAPDH into virons, we analyzed GAPDH incorporated into virions and present in CEM/LAV-1 cells and the HIV-1-noninfected cell line CEM cells using 2D gel electrophoresis and western immunoblotting. We found that five isozymes of GAPDH (spot i (pI 7.61), spot ii (pI 7.63), spot iii (pI 8.03), spot iv (pI 8.50), and spot v (pI 9.02)) were inside the virions (Figure 1C). Surprisingly, regardless of HIV-1 infection, in the cellular pool, GAPDH was identified as six isozymes that contain an additional isozyme (spot vi (pI 8.68)) with five isozymes packaged inside the virions (Figure 1D). These results suggest that the GAPDH isozymes (pI 7.61, 7.63, 8.03, 8.50, and 9.02) might be selectively packaged inside the virions. Therefore, we focused primarily on the characterization of GAPDH present within the virions.

### GAPDH-packaging-defective virus exhibits higher infectivity than control viruses

**Figure 2:**
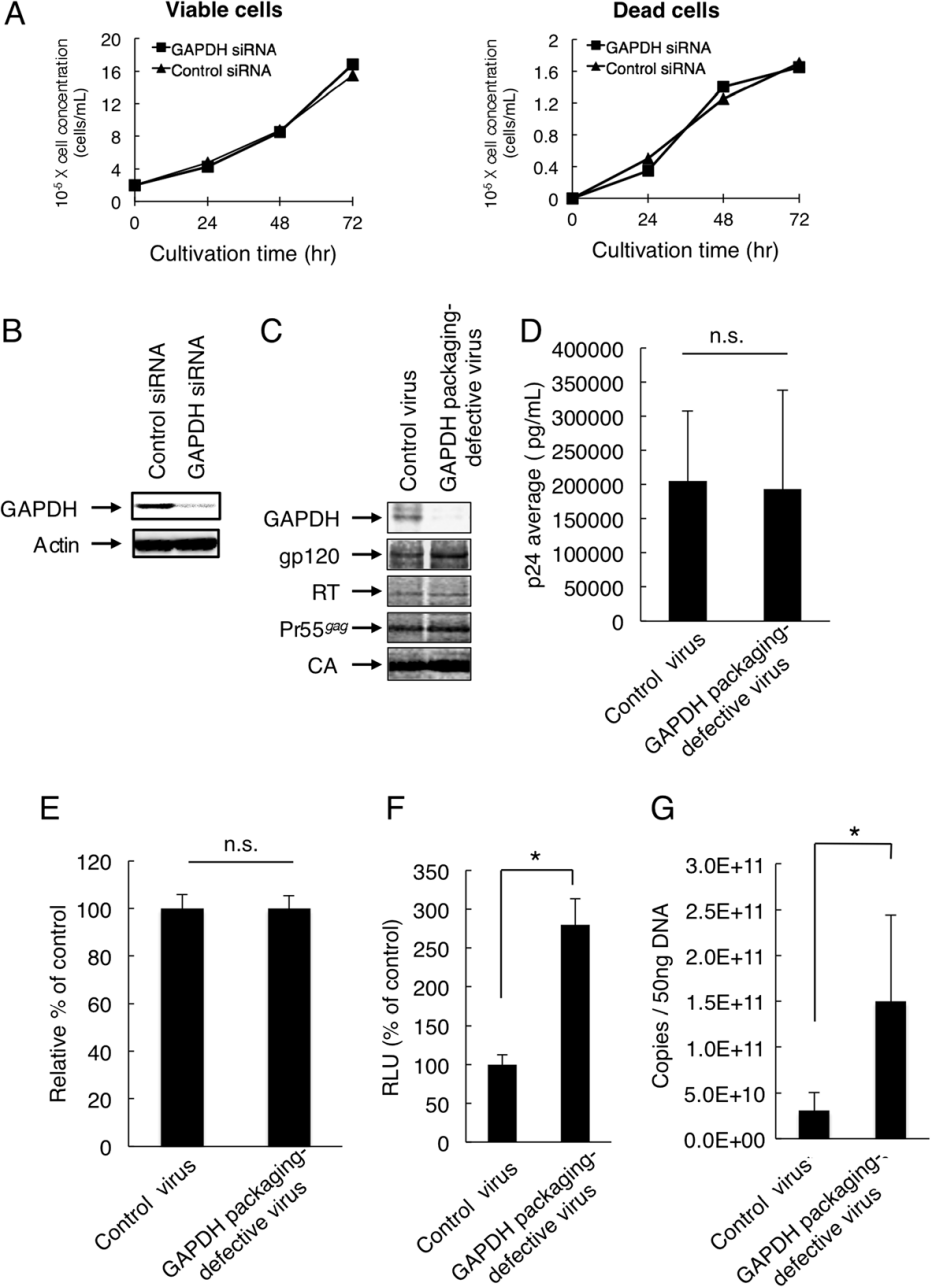
**Effects of GAPDH packaging defect inside virions on HIV**-**1 replication.** (**A**) Effects of GAPDH siRNA treatment on CEM/LAV-1 cells. Viable cells (left) and dead cells (right) were assessed by trypan blue staining. (**B**) GAPDH siRNA knockdown efficiency is confirmed by western immunoblotting. Expression of GAPDH was analyzed in cell lysates from CEM/LAV-1 cells transfected with GAPDH or control siRNA 72 h after transfection. (**C**) Effects of GAPDH siRNA on incorporation of GAPDH and HIV-1 proteins inside virions. The incorporation of GAPDH, gp120, RT, Pr55^*gag*^, or CA was analyzed by western immunoblotting of lysates from viruses produced from CEM/LAV-1 cells transfected with GAPDH or control siRNA. The anti-GAPDH antibody and HIV-1-positive plasma were used for western immunoblotting. (**D**) Effects of GAPDH siRNA on virus release in CEM/LAV-1 cells transfected with GAPDH or control siRNA. The virus release in culture supernatant was directly determined by p24 ELISA. The mean values of at least three independent experiments are shown. (**E**) Effects of GAPDH siRNA on the incorporation of HIV genomic RNA into viral particles. The level of genomic RNA in the control virus (normalized to RT activities) was set as 100%. (**F**) Infectivity of GAPDH-packaging-defective virus. Infectivity was evaluated on the basis of the luciferase activity in lysates of TZM-bl cells. The value in the control experiment was set as 100%. The mean values of at least three independent experiments are shown. (**G**) Effect of defect in GAPDH packaging on reverse transcription in TZM-bl cells. The early strong-stop DNA products were determined by quantitative real-time PCR analysis as described in “Methods”. The significance of difference (Student’s *t*-*test*) is indicated as follows: *, *p*<0.01; n.s., not significant. The mean values of at least three independent experiments are shown. The *error bars* denote the standard deviation.

To determine whether a decrease in the packaging level of virion-associated GAPDH affects HIV-1 infectivity, we transfected CEM/LAV-1 cells with GAPDH siRNA or control siRNA, and observed no toxic effects on cell viability and proliferation (Figure 2A). As shown in Figures 2B and C, GAPDH siRNA specifically decreased GAPDH level not only in CEM/LAV-1 cells without altering the level of actin but also in the virions without altering the levels of gp120, HIV-1 RT, Pr55^*gag*^, and CA protein. Furthermore, p24 ELISA indicated that the GAPDH-packaging-defective virus did not show a defect in viral release (Figure 2D) and the incorporation of HIV genomic RNA into viral particles (Figure 2E). However, the GAPDH-packaging-defective virus showed an increased infectivity (Figure 2F, *p*< 0.01) compared with the control virus. To further clarify the relationship between the increased infectivity and the reverse transcription efficiency of the GAPDH-packaging-defective virus in TZM-bl cells, we carried out quantitative real-time PCR analysis to quantify the early strong-stop DNA of reverse transcription. The GAPDH-packaging-defective virus showed a significant increase in the level of the early strong-stop form of the viral cDNA product (Figure 2G, *p*<0.01). These results suggest that the enhanced infectivity of the GAPDH-packaging-defective virus is linked to a higher efficiency of the early reverse transcription process.

### A suppressed packaging of GAPDH is accompanied by an enhanced packaging of LysRS and tRNA^Lys3^ in the virions

**Figure 3:**
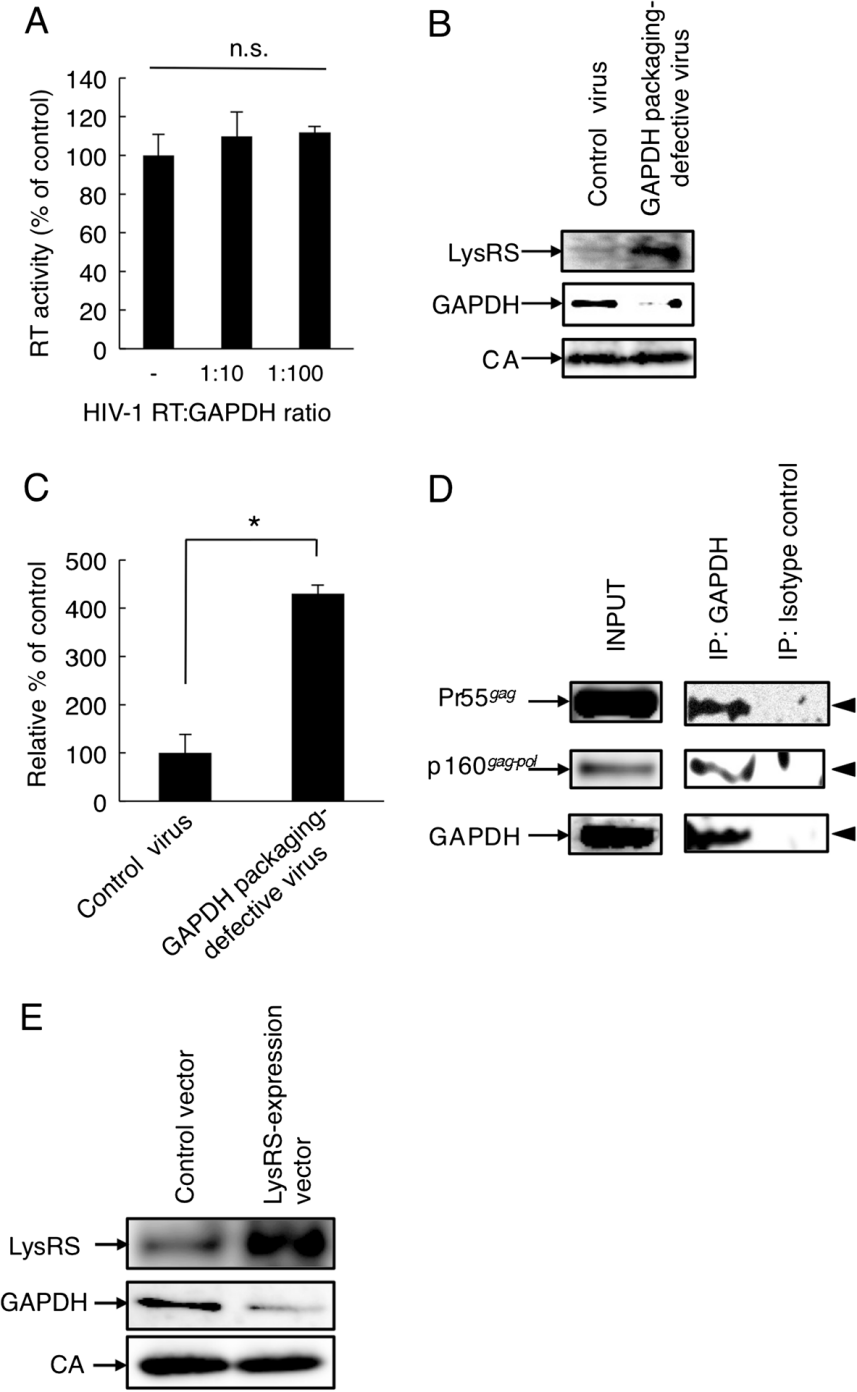
**Packaging of LysRS and tRNA**^**Lys3**^**is regulated by GAPDH.** (**A**) Effect of GAPDH on enzymatic activity of HIV-1 RT. RT activity assay was performed as described in “Methods”. The value in the control experiment was set as 100%. The activity in the presence of GAPDH (RT:GAPDH ratio= 1:10 or 1:100) is shown as the activity relative to that of the control. The mean values of at least three independent experiments are shown. Packaging of (**B**) LysRS and (**C**) tRNA^Lys3^ in GAPDH-packaging-defective virus. (**B**) LysRS and GAPDH were detected by western immunoblotting using anti-LysRS and anti-GAPDH antibodies in lysates from viral particles produced from CEM/LAV-1 cells transfected with GAPDH or control siRNA. (**C**) Incorporated tRNA^Lys3^ level was determined by reverse transcription quantitative PCR analysis as described in “Methods” and normalized by viral genomic RNA level. The amount of tRNA^Lys3^ in the control virus was set as 100%. The mean values of at least three independent experiments are shown. (**D**) Interaction of GAPDH with Pr55^*gag*^ and p160^*gag*-*pol*^. GAPDH was immunoprecipitated from the clarified lysate from CEM/LAV-1 cells with the anti-GAPDH antibody. The precipitated proteins were analyzed by western immunoblotting using the indicated antibodies to the proteins shown (anti-p24 antibody for Pr55^*gag*^ and anti-RT antibody for p160^*gag*-*pol*^), and were visualized by enhanced chemiluminescence analysis. The significance of difference (Student’s *t*-*test*) is indicated as follows: *, *p*<0.01; n.s., not significant. The *error bars* denote the standard deviation. (**E**) The packaging of GAPDH in the enhanced-LysRS-packaging virus. The packaging efficiencies of LysRS and GAPDH were analyzed in lysates from viral particles produced from HEK293 cells cotransfected with pNL-CH and either the LysRS expression or control vector.

To investigate why the GAPDH-packaging-defective virus showed a significant increase in the level of the early (R/U5) form of the viral cDNA product, *in vitro* HIV-1 RT activity was quantified in the presence of GAPDH. In RT assay, the ability of HIV-1 RT to synthesize DNA is utilized using hybrid poly(A)-oligo (dT)_15_ as a template and the primer, in the presence of recombinant GAPDH. The assays showed that GAPDH did not allosterically inhibit the activity of HIV-1 RT (Figure 3A). Gabor *et al*. [4] reported that a greater packaging of LysRS into virions is accompanied by increased tRNA^Lys3^ packaging, initiation of reverse transcription, and increased infectivity of the viral population. Therefore, we next investigated whether a decrease in virion-associated GAPDH level results in an enhanced packaging of LysRS and tRNA^Lys3^ inside the virions. Figures 3B and C show the effect of GAPDH siRNA treatment on the packaging of LysRS and tRNA^Lys3^ inside the virions. Western immunoblotting and quantitative real-time PCR analysis demonstrated that the reduction in virion-associated GAPDH level correlates with an about 1.5-fold increase in viral LysRS packaging level (Figure 3B) and an about 4-fold increase in viral tRNA^Lys3^ packaging level (Figure 3C, *p*< 0.01). Because Javanbakht *et al*. [20] and Mak *et al*. [21] further reported that LysRS packaging requires the viral Pr55^*gag*^ and tRNA^Lys3^ packaging additionally requires the p160^*gag*-*pol*^ precursor, we examined whether GAPDH could interact directly with Pr55^*gag*^ and p160^*gag*-*pol*^. Immunoprecipitation assay using an anti-GAPDH antibody showed that GAPDH directly interacts with Pr55^*gag*^ and p160^*gag*-*pol*^ (Figure 3D). Furthermore, we concluded that GAPDH does not directly interact with LysRS because no sufficient signal was detected (data not shown). However, the overexpression of LysRS in HIV-1-producing cells results in an increase in LysRS packaging level in HIV particles but a decrease in GAPDH packaging level (Figure 3E). These results suggest that the incorporation of GAPDH inside virions may contribute to the suppression of initiation of reverse transcription owing to the suppression of the packaging of LysRS and tRNA^Lys3^ inside the virions.

### An enhanced GAPDH packaging decreased viral infectivity

**Figure 4:**
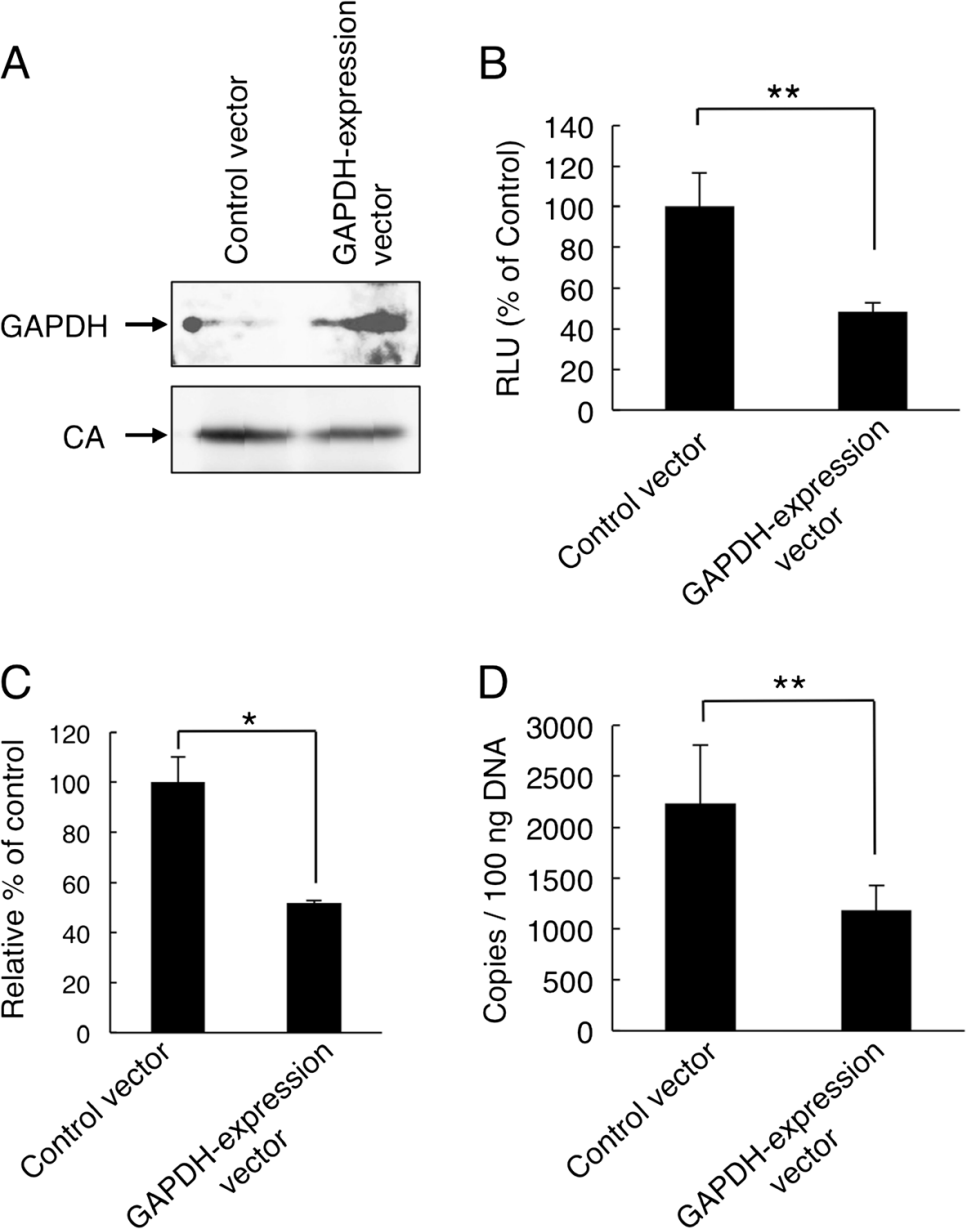
**Effects of enhanced GAPDH packaging inside virions on HIV**-**1 replication.** (**A**) Enhanced GAPDH packaging inside virions. The packaging efficiency of GAPDH was analyzed in lysates from viral particles produced from HEK293 cells cotransfected with pNL-CH and either the GAPDH expression or control vector. (**B**) Infectivity of enhanced-GAPDH-packaging virus. TZM-bl cells were incubated with an equal amount (2 ng of p24 antigen) of the enhanced-GAPDH-packaging virus or the control virus. Infectivity was evaluated as described in “Methods”. The mean values of at least three independent experiments are shown. (**C**) Quantification of tRNA^Lys3^ in enhanced-GAPDH-packaging virus. Incorporated tRNA^Lys3^ level was determined by reverse transcription quantitative PCR analysis as described in “Methods” and normalized by viral genomic RNA level. The amount of tRNA^Lys3^ in the control virus was set as 100%. The mean values of at least three independent experiments are shown. (**D**) Enhanced GAPDH packaging inside virions decreased reverse transcription efficiency in PMBCs. PBMCs were incubated with an equal amount (20 ng of p24 antigen) of the enhanced-GAPDH-packaging virus or the control virus. Reverse transcription products (R/gag DNA products) were determined as described in “Methods”. The significance of difference (Student’s *t*-*test*) is indicated as follows: **, *p*<0.05. The mean values of at least three independent experiments are shown. The *error bars* denote the standard deviation.

We next investigated whether the virus produced from HEK293 cells with high GAPDH expression levels showed decreased infectivity. We cotransfected HEK293 cells with the HIV-1 expression plasmid pNL-CH and either a GAPDH expression vector or a control vector. As shown in Figure 4A, the GAPDH expression vector increased the GAPDH level inside the virions. As expected, the enhanced-GAPDH-packaging virus decreased the viral infectivity in TZM-bl cells because the enhanced GAPDH packaging decreased tRNA^Lys3^ packaging (Figures 4B and C). Furthermore, we examined whether the enhanced packaging of GAPDH suppressed the viral replication in primary PBMCs. Interestingly, the enhanced-GAPDH-packaging virus showed a significant decrease in the level of the R/gag DNA product in PBMCs (Figure 4D). These results suggest that an enhanced GAPDH packaging decreased viral infectivity.

## Discussion

Current drugs against HIV-1 inhibit the function of viral enzymes, namely, RT, integrase, and protease. While these are especially proving useful in combination therapy, drug resistance could still be generated and appears to result from viral replication that allows the error-prone reverse transcription step. Therefore, future directions towards a more effective therapy for HIV-1 infection will rely on the development of novel therapeutic strategies rather than conventional strategies, which target only viral proteins. HIV-1 exploits multiple host proteins during infection, suggesting that the possibility of interrupting virus-host interactions may be an important pathway for the development of antiviral therapies. In concept, this type of antiviral agent developed through this pathway could minimize the generation of drug-resistant mutants because the virus must maintain the ability to interact with the relatively immutable host proteins. Thus, the interaction between the host and viral proteins may offer some potential applications for therapies against HIV-1.

**Figure 5:**
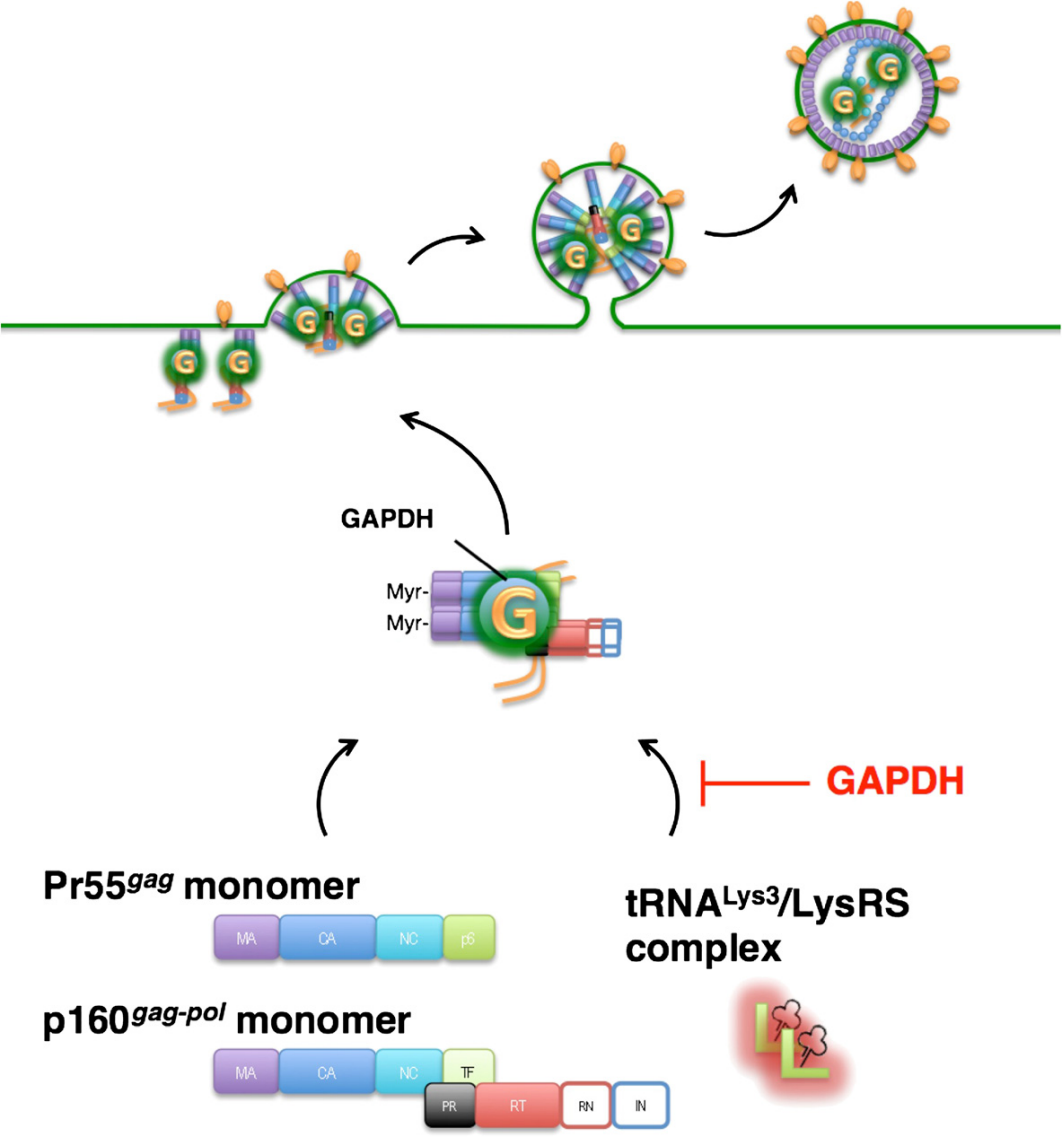
**Schematic illustration showing the mechanism by which GAPDH negatively regulates HIV-1 infection.** A single copy of each molecule is shown for simplicity. As described in detail elsewhere [20,26,27], a Pr55^*gag*^/p160^*gag*-*pol*^/viral RNA complex interacts with a tRNA^Lys3^/LysRS complex. Although the actual conformation of the Pr55^*gag*^/p160^*gag*-*pol*^/viral RNA complex is still unclear, findings of this study suggest that GAPDH and tRNA^Lys3^/LysRS complex may compete with each other for interaction with the binding site within Pr55^*gag*^ and p160gag-pol.

Proteomic analysis of HIV-1 particles is a powerful tool to identify not only novel host proteins that are packaged during viral budding but also post-translational modifications of host and viral proteins in the virions. In our previous study, three isoforms of cyclophilin A associated with HIV-1 and the post-translational modifications such as *N*-acetylation of cyclophilin A and formylation of CA protein were identified [22,23]. In this study, GAPDH isozymes with different isoelectric points were detected in the virions. Although Chertova *et al*. [24] and Ott *et al*. [25] also reported the GAPDH incorporation into HIV-1 virions produced from lymphocytes and macrophages, the virological significance of GAPDH has remained unclear. To gain insight into the role of GAPDH in viral replication, we prepared the GAPDH-packaging-defective virus by suppressing GAPDH expression by RNA interference in CEM/LAV-1 cells, and investigated the phenotypic and functional property changes of the virus. Here, we demonstrated that the suppression of GAPDH packaging increased the efficiencies of LysRS and tRNA^Lys3^ packaging into the virions, resulting in the increase in infectivity, and that GAPDH directly interacted with Pr55^*gag*^ and p160^*gag*-*pol*^, which are required for the packaging of the LysRS and tRNA^Lys3^ complex. Javanbakht *et al*. [20,26] reported that the interaction between the *gag* protein and LysRS is dependent on the last 54 amino acids of the CA C-terminal domain of the *gag* protein and amino acids 208–259 of LysRS and that the packaging of tRNA^Lys3^ requires interaction with LysRS. Furthermore, Khorchid *et al*. [27] reported that the interaction between p160^*gag*-*pol*^ and tRNA^Lys3^ involves the thumb domain (TH) sequences in RT. From these findings, Kleiman *et al*. postulated a conventional model for the formation of a tRNA^Lys3^ packaging complex, in which a Pr55^*gag*^/p160^*gag*-*pol*^/viral genomic RNA complex interacts with a tRNA^Lys3^/LysRS complex. On the other hand, our findings suggest that GAPDH and tRNA^Lys3^/LysRS complex may compete with each other for the interaction to the binding domain within the Pr55^*gag*^/p160^*gag*-*pol*^/viral genomic RNA complex (Figure 5). Taken together, these results indicate that GAPDH negatively regulates HIV-1 infection, and small molecules that reconstitute the binding mode of GAPDH to Pr55^*gag*^ and p160^*gag*-*pol*^ may interrupt Pr55^*gag*^-LysRS or p160^*gag*-*pol*^-LysRS interactions.

## Conclusions

We conclude that GAPDH is incorporated into virions and that this negatively regulates HIV-1 infection by reducing LysRS and tRNA^Lys3^ packaging efficiency. Elucidating the role of host proteins in HIV-1 infection is a critical issue and may reveal novel mechanisms of pathogenesis that may lead to the discovery of new antiviral targets.

## Methods

### Cell culture

A chronically HIV-1_LAV-1_-infected T-cell line (CEM/LAV-1) and HEK293 cells were maintained at 37°C in RPMI-1640 and DMEM supplemented with 10% fetal calf serum (FCS) containing 100 IU/ml penicillin and 100 μg/ml streptomycin in 5% CO_2_. TZM-bl cells were obtained from the NIH AIDS Research and Reference Reagent Program. This is a HeLa cell clone that was engineered to express CD4 and CCR5 and contains integrated reporter genes for firefly luciferase and *E*. *coli* β-galactosidase under the control of an HIV-1 LTR, permitting sensitive and accurate measurements of infection. PBMCs were isolated using Ficoll-Paque (Pharmacia Biotech, Piscataway, NJ) density gradient centrifugation. For concanavalin A (ConA) stimulation of PBMCs, 2 × 10^6^ cells were incubated for 24 h in RPMI-1640 containing 10% FCS, 4 mM l-glutamine, 2 mM sodium pyruvate, 50 μM 2-ME, and 25 μg/mL ConA (Sigma, St. Louis, MO). After stimulation with ConA, PBMCs were further incubated for 72 h in RPMI-1640 containing 10% FCS, 4 mM l-glutamine, 2 mM sodium pyruvate, 50 μM 2-ME, and 100 U/ml IL-2.

### Plasmids

The coding regions of human GAPDH cDNAs (GenBank™ accession number M33197.1) were amplified by PCR using the primers GF (5′-AGGATCCGCCATGGGGAAGGTGAAGGTCGG-3′) and GR (5′-GCCCACATGGCCTCCAAGGAGGATATCA-3′). The regions were cloned into the *Eco*RV and *Bam*HI sites of the pcDNA™3.1D/V5-His-TOPO® vector (Life Technologies Corporation). The coding regions of the human LysRS cDNAs (GenBank™ accession number NM_001130089) were amplified by PCR using the primers LF (5′-AGGTACCGCCATGTTGACGCAAGCTGCTGTAAGG-3′) and LR (5′-ATTCGAAGACAGAAGTGCCAACTGTTG-3′). The regions were cloned into the *Kpn*I and *Bst*BI sites of the pcDNA™3.1D/V5-His-TOPO® vector (Life Technologies Corporation).

### Viruses

Infectious HIV-1_LAV-1_ stocks were prepared from culture supernatants of CEM/LAV-1 cells [28]. Briefly, the supernatant from the culture medium of CEM/LAV-1 cells was filtered through a 0.22-μm-pore size disposable filter and then centrifuged at 43,000 × *g* for 3 h at 4°C. The obtained pellet was resuspended in PBS(−) (0.02% KH_2_PO_4_, 0.29% Na_2_HPO_4_·12H_2_O, 0.8% NaCl, 0.02% KCl) and then centrifuged at 100,000 × *g* for 1 h at 4°C. The resulting pellet was resuspended in PBS(−) and used as an infectious HIV-1_LAV-1_ stock. GAPDH-packaging-defective viruses were prepared by GAPDH siRNA treatment of CEM/LAV-1 cells. CEM/LAV-1 cells (5 × 10^5^ cells) were transfected with 50 nM validated commercially available Silencer GAPDH siRNA (Catalog #:AM4605, the siRNA sequence is not released, Life Technologies Corporation) or 50 nM control siRNA. After 24 h, the CEM/LAV-1 cells were washed and cultured for 48 h with antibiotic-free RPMI containing 10% FCS. The virus-containing supernatant was collected 72 h after transfection. On the other hand, the enhanced-GAPDH- or enhanced-LysRS-packaging virus was prepared by cotransfection of HEK293 cells (5 × 10^5^ cells) with pNL-CH [29] and either the pcDNA™3.1D-GAPDH-V5-His-TOPO® (GAPDH expression vector) or pcDNA™3.1D-LysRS-V5-His-TOPO® (LysRS expression vector). pNL-CH, derived from the pNL4-3 clone of HIV-1, contains a silent T-to-C mutation at nucleotide 2600 to introduce an *Rsr*II restriction enzyme site near the 5′ end of *pol*.

### CD45 affinity depletion

HIV-1_LAV-1_ was collected from CEM/LAV-1 culture medium and prepared according to previously described methods [28], with slight modifications. The supernatant from the culture medium of CEM/LAV-1 cells was filtered through a 0.22-μm-pore size disposable filter and then centrifuged at 43,000 × *g* for 3 h at 4°C. The obtained pellet was resuspended in PBS(−) and then centrifuged at 100,000 × *g* for 1 h at 4°C. The resulting pellet was resuspended in PBS(−). To remove CD45-positive contaminating particles such as microvesicles and exosomes from HIV-1 preparations, HIV-1 preparations containing microvesicles and exosomes were incubated with 2 μl of prewashed CD45 microbeads (Miltenyi Biotech, Auburn, CA) per 1 μg of total protein overnight on ice according to previously described methods [19], with slight modifications. In Figure 1B, forty micrograms (total protein) of purified HIV-1_LAV-1_ produced from CEM/LAV-1 cells (starting sample) was subjected to the CD45 depletion procedure. The mixture of sample-CD45 microbeads was then applied to a washed and magnetized Macs separation column (Miltenyi Biotech). The flow-through fraction was collected to obtain the CD45-depleted fraction. The column was sufficiently washed with PBS(−). To recover the contaminating particles retained on the beads as the CD45-containing fraction, preheated (95°C) SDS gel loading buffer (62.5 mM Tris–HCl, 2% SDS, 10% glycerol (pH 6.8), and 10% 2-mercaptoethanol) was applied on to the column matrix. To compare the CD45 level between the “CD45-depleted” and “CD45-containing” lanes in Figure 1B, equal amounts of samples by volume were loaded on the gel in accordance with the method of Ott *et al*. (*J*. *Virol*. 2003, 77:12699–12709.). Twenty microliter portions of fractions equivalent to the starting sample (by volume) were analyzed by CD45 immunoblotting (Figure 1B).

### Two-dimensional electrophoresis analysis

For 2D gel electrophoresis, the viral pellets derived from HIV-1_LAV-1_ were boiled for 5 min and lysed with 100 μl of lysis buffer (9.5 M urea, 2% (w/v) NP-40, 2% ampholine (pHs 3–10), and 5% 2-mercaptoethanol). Furthermore, CEM/LAV-1 or CEM cells were also lysed with the same lysis buffer. An aliquot of the lysate (1 mg of proteins for in-gel digestion, or 300 μg of proteins for western immunoblotting) was subjected to 2D polyacrylamide gel electrophoresis according to the method of O’Farrell [30].

### Protein identification by mass spectrometry

The gels obtained after 2D gel electrophoresis were stained with SYPRO® Ruby (Invitrogen). SYPRO® Ruby-stained gel pieces were excised from the gel and in-gel-digested with trypsin, as previously described [28]. For the analysis in the MS and MS/MS mode, 1 μl of the matrix (alpha-cyano-4-cinnamic acid saturated in 40% acetonitrile, 0.1% trifluoroacetic acid) per microliter of the tryptic digest was deposited on a ground steel target plate. Analysis was conducted using a MALDI-TOF/TOF UltrafleXtreme (Bruker Daltonics Inc.). Database search was performed on the NCBInr database using Mascot software (Matrix Science, London, UK).

### One-dimensional electrophoresis and western immunoblotting

SDS-polyacrylamide gel electrophoresis was performed on 5-20% polyacrylamide gels according to the method of Laemmli [31]. Electrophoresis was carried out at 20 mA per gel for 90 min. Proteins were then transferred onto a PVDF membrane at 0.8 mA/cm^2^ for 70 min. The membrane was saturated in 5% skimmed milk powder in TBS (10 mM Tris–HCl (pH 7.5), 0.5 M NaCl) and incubated for 4 h with each primary antibody. After washing in TBS-T (10 mM Tris–HCl (pH 7.5), 0.5 M NaCl, 0.5% Tween 20), the membrane was further incubated for 1 h with each secondary antibody, and immunoreaction products were visualized using a SuperSignal® West Pico Chemiluminescent substrate (Thermo Fisher Scientific Inc.).

### Suppression of GAPDH packaging by siRNA and virus release assay

To examine the cytotoxicity induced by siRNA treatment, trypan blue dye exclusion assay was carried out. GAPDH siRNA knockdown efficiency in CEM/LAV-1 cells, which were transfected with GAPDH or control siRNA, 72 h after transfection was confirmed by western immunoblotting using an anti-GAPDH antibody (SIGMA). The packaging efficiency of GAPDH, LysRS, and viral structural proteins into virions was monitored by western immunoblotting using an anti-GAPDH antibody, an anti-LysRS antibody (Cell Signaling Technology), and HIV-1-positive plasma (a kind gift from Dr. Shuzo Matsusita, AIDS Research Institute, Kumamoto University, Kumamoto, Japan). The release of the GAPDH-packaging-defective virus was directly monitored by p24 ELISA (ZeptoMetrix Corporation) as the amount of the CA protein in culture supernatants of CEM/LAV-1 cells transfected with GAPDH or control siRNA.

### Determination of HIV-1 infectivity

The infectivity of the GAPDH-packaging-defective virus or enhanced-GAPDH-packaging virus was quantified using TZM-bl cells, which express a luciferase gene and a beta-galactosidase gene under the control of the HIV-1 LTR promoter [32]. TZM-bl cells (4 × 10^5^ cells) were incubated with the GAPDH-packaging-defective, enhanced-GAPDH-packaging, or control virus with 20 μg/ml DEAE dextran for 2 h at 37°C and then cultured in DMEM supplemented with 10% FCS (200 μl) for 48 h. The cells were fixed, and the HIV-1 infection of TZM-bl cells was determined by measuring the luciferase activity in cell lysates.

### Quantitative analysis of HIV-1 reverse transcription during acute infection

*De novo*-synthesized HIV-1 cDNA was analyzed using the protocol of Ikeda *et al*. [33]. Briefly, the TZM-bl cells or PBMCs (1 × 10^6^ cells) were infected with either the GAPDH-packaging-defective virus or the enhanced-GAPDH-packaging virus and incubated for 4 h at 37°C. The cells were washed with PBS(−), incubated for 20 h at 37°C, washed with PBS(−), and further incubated for 5 min at 37°C in PBS(−) containing 0.25% trypsin. After trypsinization, the cells were washed twice with PBS(−) and then digested in 200 μl of digestion buffer (10 mM Tris–HCl (pH 8.0), 150 mM NaCl, 10 mM EDTA, 0.1% SDS, 100 μg/ml proteinase K) for 2 h at 50°C. After digestion, proteinase K was heat-inactivated for 10 min at 95°C. To measure the amounts of early reverse transcription products in GAPDH-packaging-defective virus infection, the sample was subjected to quantitative real-time PCR with a primer pair specific for the R/U5 region (M667, 5′-GGCTAACTAGGGAACCCACTG-3′; AA55: 5′-CTGCTAGAGATTTTCCACACTGAC-3′). To further measure the amounts of late reverse transcription products in enhanced-GAPDH-packaging virus infection, a primer pair specific for the R/gag region (M667, 5′-GGCTAACTAGGGAACCCACTG-3′; M661, 5′-CCTGCGTCGAGAGAGCT CCTCTGG-3′) was used. Because the primer pair R/U5 used detects both early and late products, the following computation was used to determine the amount of early strong-stop DNA: the copy number of strong-stop DNA=R/U5 DNA-R/gag DNA copies.

### RT activity assay

To investigate whether GAPDH could allosterically reduce RT activity, recombinant GAPDH (Sigma-Aldrich Co., LLC.) and a reverse transcription assay kit (F. Hoffmann-La Roche Ltd.) were used in this assay. Briefly, the solution (46 mM Tris–HCl, 266 mM potassium chloride, 27.5 mM magnesium chloride, 9.2 mM DTT, digoxigenin (DIG)-labeled dUTP, biotin-labeled dUTP, dTTP, and poly(A) × oligo(dT)_15_ template/primer hybrid) was added to the reaction tube containing the HIV-1 RT standard preincubated for 1 h with recombinant GAPDH at a ratio of 1:10 or 1:100, and then incubated for 1 h at 37°C. After finishing the RT reaction, the reaction mixture was transferred to streptavidin-coated microtitre plates. DIG-labeled DNA was detected with an anti-DIG-POD conjugate, reacted with 2,2-azino-di(3-ethylbenzthiazoline) sulfonic acid, and quantitated by measuring OD at 405/490 nm. The HIV-1-RT inhibition assay was performed as described in the kit protocol.

### Quantification of viral genomic RNA and tRNA^Lys3^ packaging levels in virions

Both viral genomic RNA and tRNA^Lys3^ were collected using a QIAamp® Viral RNA Mini kit (Qiagen) or Nucleo Spin® miRNA (Macherey-Nagel). Genomic RNA was reverse-transcribed using a SuperScript® VILO™ cDNA Synthesis kit and quantified using a primer pair specific for the R/gag region (M667, 5′-GGCTAACTAGGGAACCCACTG-3′; M661, 5′-CCTGCGTCGAGAGAGCTCCTCTGG-3′), or the primers SK38 (5′-ATAATCCACCTATCCCAGTAGGAGAAAT-3′) and SK39 (5′-TTTGGTCCTTGTCTTATGTCCAGAATGC-3′). On the other hand, tRNA^Lys3^ was reverse-transcribed by a SuperScript™III First-Strand Synthesis System for RT-PCR (Life Technologies Corporation) using a tRNA^Lys3^-F-primer (5′-TGGCGCCCGAACAGGGAC-3′) and then quantified by a tRNA^Lys3^-F-primer and a tRNA^Lys3^-R-primer (5′-GCATCAGACTTTTAATCTGAGGG-3′). Quantitative real-time PCR was carried out with a SsoFAST™ EvaGreen® Supermix (Bio-Rad Laboratories, Inc.); the cycling conditions were 98°C for 2 min, then 98°C for 5 sec, followed by 40 cycles of 15 sec at 60°C.

### Coimmunoprecipitation

CEM/LAV-1 cells were lysed in 350 μl of RIPA buffer (50 mM Tris–HCl (pH 7.4), 150 mM NaCl, 1% sodium deoxycholate, 0.1% SDS, 1% Triton X-100). The insoluble material was pelleted, and the supernatant was used for coimmunoprecipitation. The supernatant was precleaned with Protein-G Sepharose® without any antibody, incubated with 10 μl of an anti-GAPDH antibody (Santa Cruz Biotechnology, INC) or an isotype control goat IgG antibody (Southern Biotechnology Associates, Inc.) for 4 h at 4°C, and further incubated with 50 μl of Protein-G Sepharose® resin slurry (50% slurry in RIPA buffer) for 4 h at 4°C. After washing with RIPA buffer, the bound proteins were eluted using the SDS gel loading buffer. The precipitated proteins were detected by western immunoblotting. Pr55^*gag*^ and p160^*gag*-*pol*^ were detected using an anti-p24 antibody (ViroGen) and an anti-RT antibody (Bio Academia), respectively.

### Enhancement of GAPDH packaging by GAPDH expression vector

Enhanced-GAPDH-packaging viruses were prepared by cotransfection of HEK293 cells with pNL-CH and the GAPDH expression vector. Enhanced GAPDH packaging efficiency in viral particles is confirmed by western immunoblotting using an anti-GAPDH antibody (SIGMA). The signal of the CA protein was used as the loading control.

### Enhancement of LysRS packaging by LysRS expression vector

Enhanced-LysRS-packaging viruses were prepared by cotransfection of HEK293 cells with pNL-CH and the LysRS expression vector. Enhanced LysRS packaging efficiency in viral particles is confirmed by western immunoblotting using an anti-LysRS antibody (Cell Signaling Technology). The signal of the CA protein was used as the loading control.

## Abbreviations

GAPDH: Glyceraldehyde 3-phosphate dehydrogenase; HIV-1: Human immunodeficiency virus type 1; RT: Reverse transcriptase; LysRS: Lysyl-tRNA synthetase; PBMCs: Peripheral blood mononuclear cells; MALDI-TOF MS: Matrix-assisted laser desorption/ionization-time-of-flight mass spectrometry; CA: Capsid; MA: Matrix.

## Competing interests

The authors have no conflicting financial interests.

## Authors’ contributions

SM conceptualized and designed the study, NK, AO, KK, NT, SS, and SM performed the study, and analyzed data; NK and SM wrote and critically read the paper. All the authors reviewed the manuscript and approved the final version.
